# Potential diagnostic markers shared between non-alcoholic fatty liver disease and atherosclerosis determined by machine learning and bioinformatic analysis

**DOI:** 10.3389/fmed.2024.1322102

**Published:** 2024-03-28

**Authors:** Lihong Wang, Wenhui He, Xilin Wang, Jianrong Wang, Xiaojuan Wei, Dongzhi Wu, Yundan Wu

**Affiliations:** ^1^Department of Pharmacy, Fuzhou Second General Hospital, Fuzhou, China; ^2^Department of Orthopedic Research Institute, Fuzhou Second General Hospital, Fuzhou, China; ^3^Department of Pharmacy, The Third Affiliated Hospital of Fujian University of Traditional Chinese Medicine, Fuzhou, China

**Keywords:** non-alcoholic fatty liver disease, atherosclerosis, *RPS6KA1*, *SERPINA3*, bioinformatics

## Abstract

**Background:**

Evidence indicates that chronic non-alcoholic fatty liver disease (NAFLD) can increase the risk of atherosclerosis (AS), but the underlying mechanism remains unclear.

**Objective:**

This study is intended for confirming key genes shared between NAFLD and AS, and their clinical diagnostic value to establish a foundation for searching novel therapeutic targets.

**Methods:**

We downloaded the Gene Expression Omnibus (GEO) datasets, GSE48452 and GSE89632 for NAFLD and GSE100927, GSE40231 and GSE28829 for AS. The progression of NAFLD co-expression gene modules were recognized via weighted gene co-expression network analysis (WGCNA). We screened for differentially expressed genes (DEGs) associated with AS and identified common genes associated with NAFLD and AS using Venn diagrams. We investigated the most significant core genes between NAFLD and AS using machine learning algorithms. We then constructed a diagnostic model by creating a nomogram and evaluating its performance using ROC curves. Furthermore, the CIBERSORT algorithm was utilized to explore the immune cell infiltration between the two diseases, and evaluate the relationship between diagnostic genes and immune cells.

**Results:**

The WGCNA findings associated 1,129 key genes with NAFLD, and the difference analysis results identified 625 DEGs in AS, and 47 genes that were common to both diseases. We screened the core *RPS6KA1* and *SERPINA3* genes associated with NAFLD and AS using three machine learning algorithms. A nomogram and ROC curves demonstrated that these genes had great clinical meaning. We found differential expression of *RPS6KA1* in patients with steatosis and NASH, and of *SERPINA3* only in those with NASH compared with normal individuals. Immune infiltration findings revealed that macrophage and mast cell infiltration play important roles in the development of NAFLD and AS. Notably, *SERPINA3* correlated negatively, whereas *RPS6KA1* correlated positively with macrophages and mast cells.

**Conclusion:**

We identified *RPS6KA1* and *SERPINA3* as potential diagnostic markers for NAFLD and AS. The most promising marker for a diagnosis of NAFLD and AS might be *RPS6KA1*, whereas *SERPINA3* is the most closely related gene for NASH and AS. We believe that further exploration of these core genes will reveal the etiology and a pathological relationship between NAFLD and AS.

## Introduction

Non-alcoholic fatty liver disease (NAFLD) is the most common chronic liver disease worldwide, and its incidence has increased from 25% in 2005 to 32% today (1), rendering it a leading chronic condition. In a study of abdominal ultrasound and coronary computed tomography angiography of 5,121 patients with neither history of coronary artery disease nor chronic alcohol consumption revealed a close correlation between the fatty liver index, NAFLD fibrosis scores, and non-calcified plaque (2). This proved that NAFLD is closely associated with noncalcified plaques, which are prone to sudden and unexpected cardiac events. In other words, NAFLD may induce the occurrence of cardiovascular diseases for instance atherosclerosis (3). Furthermore, cardiovascular complications correlate with increased risk for NAFLD (4). More focus on NAFLD is needed to reduce the risk of atherosclerotic events. The clinicopathological syndrome NAFLD is characterized by diffuse hepatocellular bulla fat, alcohol consumption, and other specific liver damaging factors, including simple hepatic steatosis and the evolution of non-alcoholic steatohepatitis (NASH), cirrhosis, and liver cancer (5, 6). The main pathological feature of liver steatosis is hepatocyte steatosis (> 5%), while NASH is inflammation and fibrosis (7). A meta-analysis has revealed significant correlation between AS and any degree of fibrosis, the severity of which amplifies this association (8). The risk of atherosclerosis increases when NAFLD progresses from simple steatosis to NASH (9).

Atherosclerosis is the deposition of apolipoprotein in the inner walls of blood vessels. This recruited various immune cells, resulting in endothelial dysfunction, which leads to a chronic inflammatory response (10). A close association between NAFLD and AS has been identified, and the increased abundance of systemic inflammatory factors such as IL6, IL-1β, TNF-α leads to further endothelial dysfunction and enhanced vascular plaque formation in patients NAFLD (11). In addition, NAFLD and AS are manifestations of metabolic syndrome in end-organ damage, as well as diseases caused by abnormal fat metabolism. Therefore, systemic inflammation, endothelial dysfunction, and altered lipid metabolism might be mechanisms through which NAFLD increases the risk of AS. With the wide development and application of bioinformatics, WGCNA and machine learning algorithms (MLAs) can help to explore potential biological diagnostic markers between NAFLD with different stages and AS and assess immune characteristics between them using immune cell infiltration. This would contribute to the prevention and early treatment of both diseases. Here, we used WGCNA analysis and MLAs to explore potential biological diagnostic markers that differ between NAFLD with different progression and AS, and are common to both. We also and assessed immune signatures between potential markers using immune cell infiltration. Our findings should contribute to the prevention and early treatment of both diseases.

## Materials and methods

### Data processing and analysis

**FIGURE 1:**
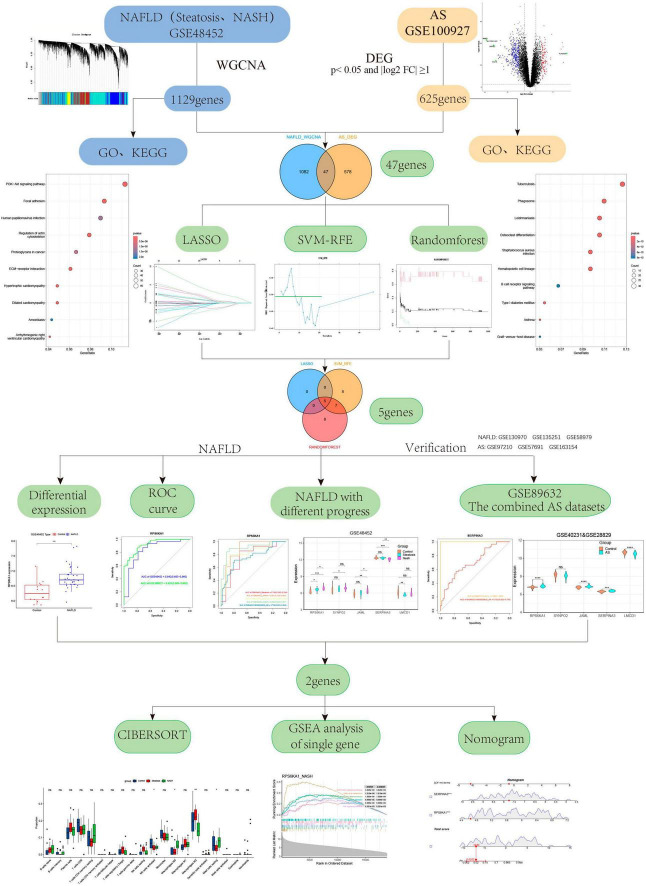
Flowchart.

We downloaded the GEO datasets^1^ GSE48452, GSE89632, GSE100927, GSE130970, GSE135251, GSE58979, GSE40231, GSE28829, GSE97210, GSE57691, and GSE163154 to determine gene expression and clinical information regarding patients with NAFLD and AS. The GSE40231 and GSE28829 were merged into one combined AS dataset through the SVA package (Supplementary Figure 1). Liver transcriptome data were derived from 32 NAFLD patients (steatosis, n = 14; NASH, n = 18) and 14 normal subjects in the GSE48452 dataset (GPL11532 platform). The GSE100927 dataset (GPL17077 platform) included 104 human peripheral artery specimens from 69 patients with AS and 35 healthy individuals. We verified the specific expression of gene signatures using the GSE89632,the combined AS dataset,GSE130970, GSE135251, GSE58979, GSE97210, GSE57691, GSE163154 datasets. All datasets were processed and analyzed using RStudio version 4.2.1. Detailed information on the GEO dataset can be found in Supplementary Table 1. Figure 1 displays the flowchart of the study. Table 1 displays the Abbreviations of the study.

**TABLE 1:** Displays the Abbreviations t of the study.

Abbreviations	Full name	Abbreviations	Full name
AS	atherosclerosis	GSEA	Gene Set Enrichment Analysis
AUC	calculated the area	KEGG	Kyoto Encyclopedia of Genes and Genomes
CCR2	chemokine receptor type 2	KLF2-eNOS	Kruppel like factor 2- endothelial nitric oxide synthase
CXCR4	C-X-C chemokine receptor type 4	LASSO	Minimum absolute contraction and selection operator
DCA	Decision curve analysis	MLAs	machine learning algorithms
DEGs	Differentially expressed genes	NAFLD	Non-alcoholic fatty liver disease
ECM	Extracellular matrix	NASH	Non-alcoholic steatohepatitis
ECs	Endothelial cells	ROC	Receiver operating characteristic
ERK5	Extracellular signal-regulated kinase 5	SVM-RFE	Support vector machine recursive feature elimination
GEO	Gene expression omnibus	VCXAM-1	Vascular cell adhesion molecule 1
GO	Gene ontology	WGCNA	Weighted gene co-expression network analysis

### Gene expression analyzed using WGCNA

Gene expression profiles in many samples can be analyzed using the WGCNA (12–14). It can cluster genes with similar expression profiles and analyze dependence between modules and particular traits or phenotypes. It is widely applied to phenotypic trait and gene association analyses among multiple groups (15). Both GSE100927 and GSE57691 datasets contain clinical information related to the progression of NAFLD. We constructed a gene co-expression network for NAFLD by the WGCNA version 1.72.1 package in R version 4.3.1. The flash cluster function in R was used analyze hierarchical clustering in NAFLD samples to identify and eliminate outliers. We then calculated an appropriate soft threshold using the pick Soft Threshold algorithm in the WGCNA package. A scale-free network was constructed to obtain various gene modules related to NAFLD using the one-step network construction function in the WGCNA package. Divide the samples into control, steatosis, and NASH, perform module phenotype correlation analysis on each module, draw a module phenotype correlation heatmap, and determine the module with the highest correlation with the target phenotype. Key NAFLD-associated gene modules (NAFLD_WGCNA), were screened by association analysis of the module data with the clinical characteristics of NAFLD.

### Difference analysis

Using R, we standardized and corrected all gene expression profile microarray data and annotated gene names using limma version 3.56.2 (16). DEGs in AS (AS_DEGs) were also obtained by limma package analysis of gene expression between AS patients and control cases. The gene threshold of AS_DEGs was p < 0.05 and |log2 FC| ≥ 1. We visualized differential data as volcano plots. Genes that were common to NAFLD_WGCNA and AS_DEGs were identified using ggvenn version 0.1.10.

### Functional enrichment analysis

We aimed to identify NAFLD and AS co-morbidities and reveal the potential biological significance of the core genes of these diseases. We therefore analyzed Gene Ontology (GO) and Kyoto Encyclopedia of Genes and Genomes (KEGG) pathways to determine the functional enrichment of NAFLD_WGCNA and AS_DEGs, respectively using the ClusterProfiler package (17) in R. Functional enrichment was confirmed at p < 0.05.

### Key genes with diagnostic value screened by MLAs

Based on the common genes in NAFLD and AS, we further screened core genes using the LASSO regression (18), SVM-RFE (19), and random forest (20). LASSO regression was applied using the glmnet package version 4.1.7 with parameters adjusted by 10-fold cross-validation. Using caret version 6.0–86, e1071 version 1.7–9, and kernlab version 0.9–29, the SVM-RFE algorithm was applied. Through five-fold cross-validation, the accuracy of the model algorithm was improved. The random forest algorithm was executed using randomForest package (version 4.7.1.1). The overlapping results of the three algorithms represented the diagnostic markers of NAFLD-associated AS. The core diagnostic markers of NAFLD-associated AS were further determined from analyses of ROC curves and gene expression.

### Verification of core genes

We verified the differential expression of key genes in control and focal tissues by the NAFLD and AS datasets GSE89632, GSE130970, GSE135251, GSE58979,and the combined AS dataset,GSE97210, GSE57691, GSE163154 datasets respectively. We established ROC curves through the pROC (21) version 1.18.4 package, calculated the AUC value, and evaluated the ability of core genes to diagnose steatosis, NASH, and AS, respectively.

### Construction of NAFLD-related AS diagnostic model

Expression profiles of the core diagnostic genes were obtained from the GSE100927 dataset. We constructed a nomogram diagnostic model (22, 23) based on gene expression, AS, and control samples to confirm the diagnostic value of these genes.

### GSEA of individual genes

We assessed core genes using GSEA (24) and the ClusterProfiler package in R. Samples of steatosis, NASH and AS were classified based on high or low expression of core genes. A series of core DEGs was determined using the limma package. We then compared KEGG pathways among the three diseases using GSEA.

### Correlations between core genes and immune cells

We explored the relationship between immune cell types with different degrees of infiltration and diagnostic markers of NAFLD and AS using CIBERSORT (25) and the preprocessCore version 1.62.1, parallel version 4.3.1, and e1071 packages in R.

### Statistical analysis

Statistical analysis of all data was performed using R and Wilcoxon tests were used for analyzing differential gene expression. Differences among the control, steatosis, and NASH groups were compared by measuring immune infiltration and analysis of variance. More detailed statistical methods for data processing were introduced in the previous section. P values greater than 0.05 were considered statistically significant.

## Results

### Identification of co-expressed gene modules in NAFLD

**FIGURE 2:**
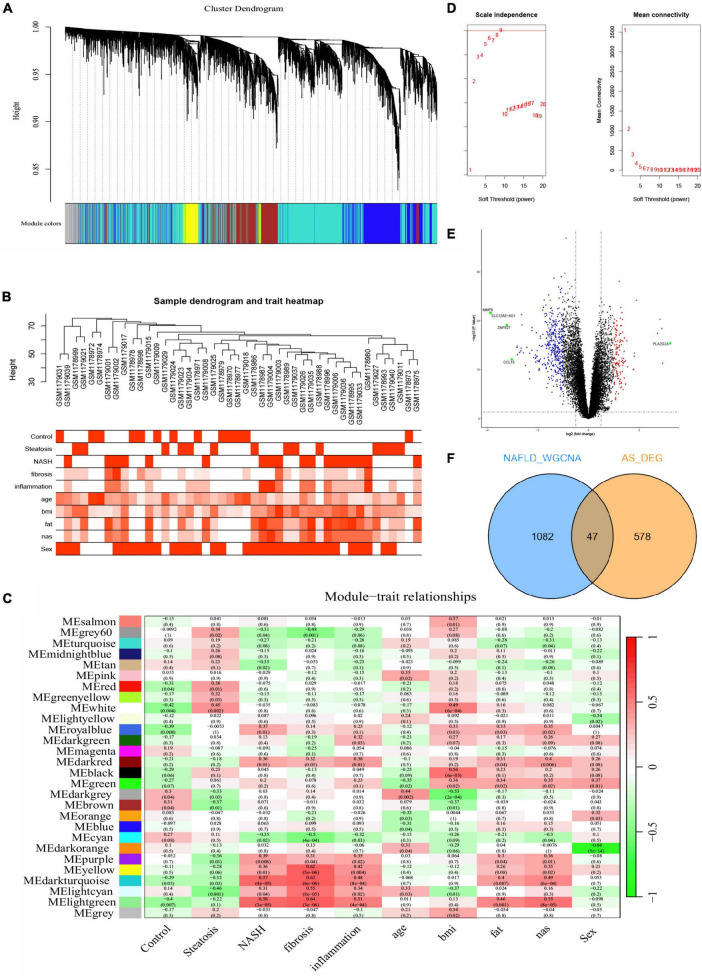
Difference analysis and WGCNA. **(A-C,E)** Construction of WGCNA co-expression network in GSE48452. **(A)** Sample clustering dendrogram. **(B)** Sample dendrogram and trait heatmap. **(C)** Soft threshold β = 9 and scale–free topological fit index (R2). **(D)** Volcano plot of DEGs in GSE100927. Red nodes indicate upregulated DEGs, blue nodes indicate downregulated DEGs, green nodes indicate *p* < 0.05 and | log_2_ FC| > 3, and black nodes indicate genes that are not significantly differentially expressed. **(E)** Heat map of module–trait correlations. **(F)** Common genes overlap in WGCNA_NAFLD and AS_DEGs.

We obtained gene expression profiles from 14 patients with steatosis, 18 with NASH, and 14 healthy individuals in the NAFLD datasets. The samples were assigned to steatosis, NASH and control groups. We ensured that the constructed scale-free network had biological significance as follows. We removed the outlier sample GSM1179010 from the NAFLD dataset, and selected nine as the soft threshold power β for the NAFLD dataset on the basis of a scale-free R2 (0.8). We also calculated GS (correlation between a gene and a sample) and MM (correlation between gene expression and genes with a modular signature; ME) to correlate these modules with clinical features. Figures 2A-D shows that the NAFLD dataset genes were clustered into 27 modules visualized in different colors. Among them, light green, dark turquoise, light cyan, yellow, and white co-expressed gene modules were closely associated with NAFLD. The light green and dark turquoise modules were most associated with NASH, and light cyan and white were most associated with steatosis. The light green, dark turquoise, light cyan, and yellow modules were related to inflammation and fibrosis. The dark turquoise, light cyan were related to fat and nas. Therefore, we selected these five NAFLD-related gene modules as targets and found 1,129 genes (NAFLD_WGCNA), which is shown in Supplementary Table 2.

### Identification of DEGs in AS

We compared DEGs between patients with AS and healthy individuals using GSE100927 datasets. With the difference analysis, 625 DEGs (Supplementary Table 2) were selected between AS and healthy controls based on combination and normalization of the microarray data (p < 0.05 and | log2 FC| ≥ 1; Figure 2E, volcano plot; Supplementary Figure 2, heatmap). The Venn diagram revealed 47 intersecting genes between AS and NAFLD (Figure 2F). Thus, these two diseases have 47 genes in common.

### Functional enrichment analysis

**FIGURE 3:**
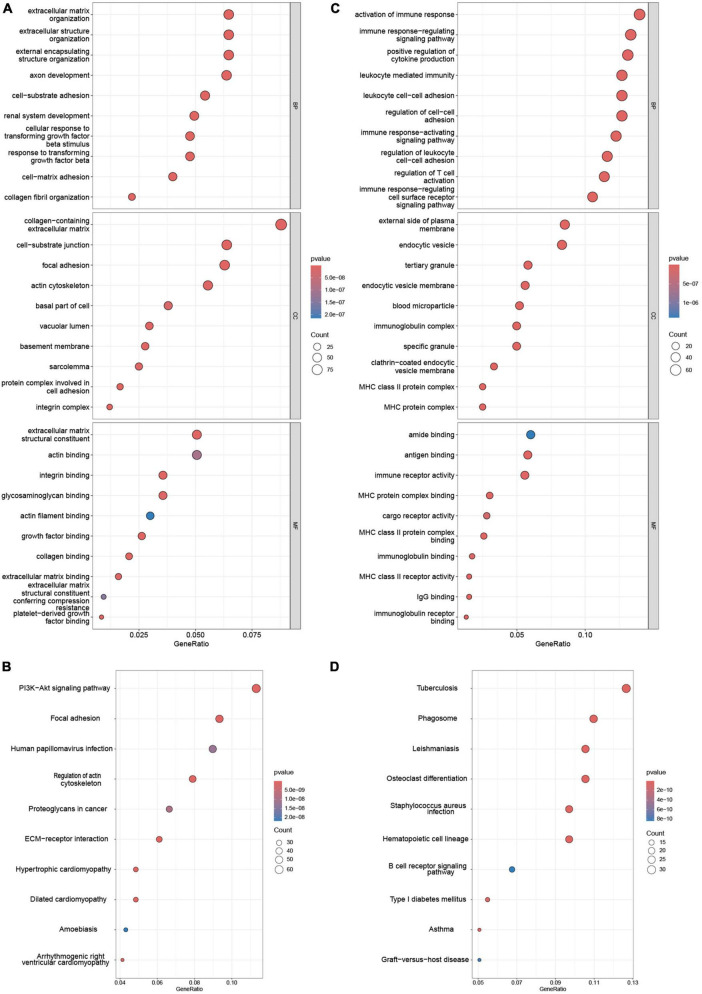
Enrichment analysis of NAFLD_WGCNA and AS_DEGs. GO **(A)** and **(B)** KEGG pathway enrichment of NAFLD_WGCNA. GO **(C)** and KEGG pathway **(D)** enrichment of AS_DEGs.

Functional enrichment of AS_DEGs and NAFLD_WGCNA was analyzed. Figures 3A-D shows that the NAFLD_WGCNA genes were concentrated in the PI3K-AKT signaling pathway, focal adhesion, ECM-receptor interaction, and other pathways. The AS_DEGs were primarily enriched in the activation of immune responses, leukocyte-mediated immunity, B cell receptor signaling, and other pathways. Collectively, these diseases appear to be mediated by inflammation and immunity.

### Machine learning

**FIGURE 4:**
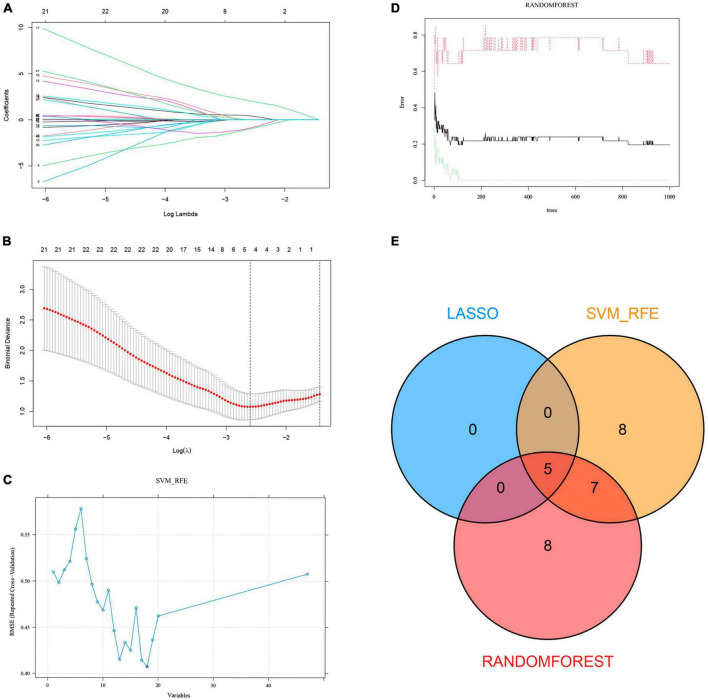
Machine learning. **(A)** LASSO coefficient profiles in GSE48452. **(B)** Log (*λ*) value determined by ten-fold cross-validation in GSE48452. Core genes **(C)** selected by SVM-RFE and **(D)** screened by Random Forest algorithm in GSE48452 and **(E)** Venn diagram.

We extracted expression data for 47 genes from the common gene expression profiles of NAFLD and AS. Five, 20, and 20 genes were respectively selected as biomarkers associated with NAFLD and AS using the LASSO, SVM-RFE, and random forest algorithms (Figures 4A-D, Supplementary Table 3 and Supplementary Figure 3). Overlapping genes derived from the three algorithms were considered diagnostic for NAFLD-AS comorbidities, and we identified them as *RPS6KA1*, *SERPINA3*, *JAML*, *SYNPO2*, and *LMCD1* (Figure 4E).

### Differentially expressed genes and ROC curves

**FIGURE 5:**
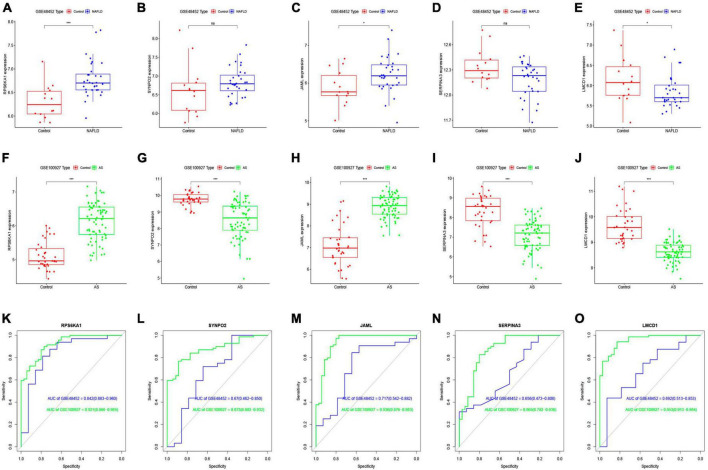
Differential expression analysis of core genes and ROC curves. Differential expression of core genes between control and NAFLD in GSE48452 **(A–E)** and between control and AS in GSE100927 **(F-J)**. ROC curves of core genes in GSE48452 and GSE100927 **(K-O)**. *P < 0.05 and ***P < 0.001. ns indicates not significant (*p* > 0.05).

We investigated the diagnostic value of the five common genes screened in clinical practice, by analyzing differences among them between the NAFLD and AS datasets. Figures 5A-J shows that three diagnostic genes significantly differed between the NAFLD and AS datasets whereas *SERPINA3* and *SYNPO2* did not. The *LMCD1* gene correlated negatively, whereas *RPS6KA1* and *JAML* correlated positively with NAFLD and AS. We then plotted ROC curves for the NAFLD and AS datasets. Figures 5K-O shows that all AUC values were > 0.6, indicating that these five genes have clinical diagnostic value.

### Five diagnostic genes and NAFLD progression

**FIGURE 6:**
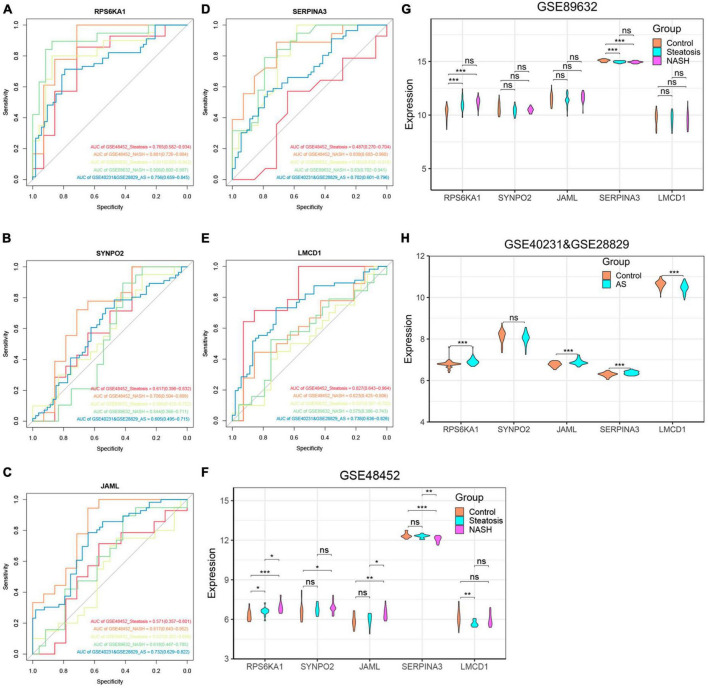
Verification of core genes. **(A-E)** Values for ROC and AUC of each hub gene among control, steatosis and NASH in GSE48452, GSE89632 and between control and AS in the combined AS dataset; **(F-H)** Differentially expressed core genes among control, steatosis and NASH in GSE48452, GSE89632 and between control and AS in the combined AS dataset. *P < 0.05, **P < 0.01, and ***P < 0.001. ns indicates not significant (*p* > 0.05).

We further explored relationships between these diagnostic genes and NAFLD progression. We found that *RPS6KA1* expression differed between the steatosis and NASH groups, with all AUCs being > 0.7, whereas *LMCD1* expression differed only in the steatosis group. The expression of *SYNP02*, *JAML*, and *SERPINA3* differed only in the NASH group, with all AUCs being > 0.7, while there was no difference in the steatosis group, with their AUC values all less than 0.7 (Figures 6A-F, Supplementary Figure 4). Therefore, we selected the *RPS6KA1* and *LMCD1* genes as diagnostic for steatosis and AS, and the *RPS6KA1 SYNP02*, *JAML* and *SERPINA3* genes as diagnostic for NASH and AS.

### Verification of core genes

We validated the accuracy of five common genes using DEGs from GSE89632 and GSE57691. Figures 6A–H and Supplementary Figure 4 shows that the expression of *RPS6KA1*, *SERPINA3*, JAML, and *LMCD1* differed in the combined AS datasets and the ROC curve results showed that they had significant value for a diagnosing AS. Similarly, we have also seen similar results on other AS datasets. The expression of *RPS6KA1* and *SERPINA3* differed in the steatosis and NASH groups in the GSE89632 and GSE58979 dataset, with AUCs being > 0.7. These results indicated that *RPS6KA1* and *SERPINA3* have significant value for diagnosing NAFLD (steatosis and NASH). Therefore, *RPS6KA1* might be a promising marker for diagnosing NAFLD and AS, whereas *RPS6KA1* and *SERPINA3* might be promising for diagnosing NASH and AS.

### Construction of diagnostic model for NAFLD-related AS

**FIGURE 7:**
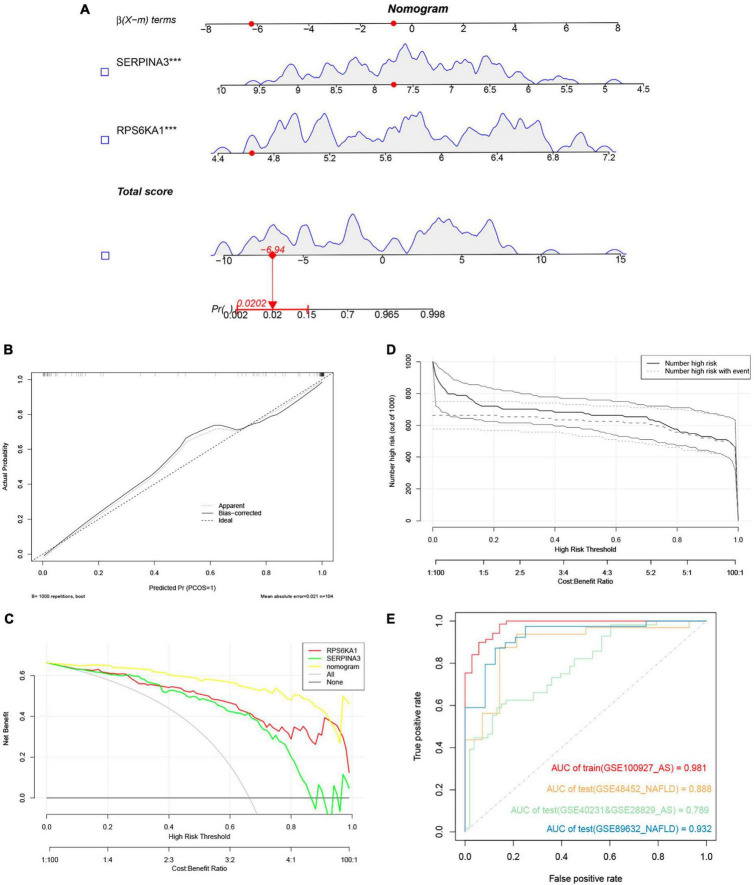
Nomogram model. **(A)** Nomogram model predicting AS based on two hub genes in GSE100927. The multiple regression analysis results between multiple predictive factors and the dependent variable, ***P < 0.001. **(B)** Calibration curves for nomogram. **(C)** DCA of nomogram and each hub gene. **(D)** Clinical impact curve of nomogram. **(E)** Validation of nomograms using ROC curves.

We evaluated the performance of the prediction model by basing a nomogram on the diagnostic genes, *RPS6KA1* and *SERPINA3*, and using calibration, decision curve analysis (DCA), and clinical impact curves (Figures 7A-D). The ROC curve in Figure 7E indicates that the nomogram has powerful diagnostic value for NAFLD-associated AS. The calibration curves in Figure 7C indicated that the prediction probability of the nomogram diagnostic model was similar to that of the ideal model, and the DCA in Figure 7D showed that NAFLD-associated AS diagnoses were more beneficial when based on the nomogram model rather than on a single gene.

### GSEA analysis of single gene

**FIGURE 8:**
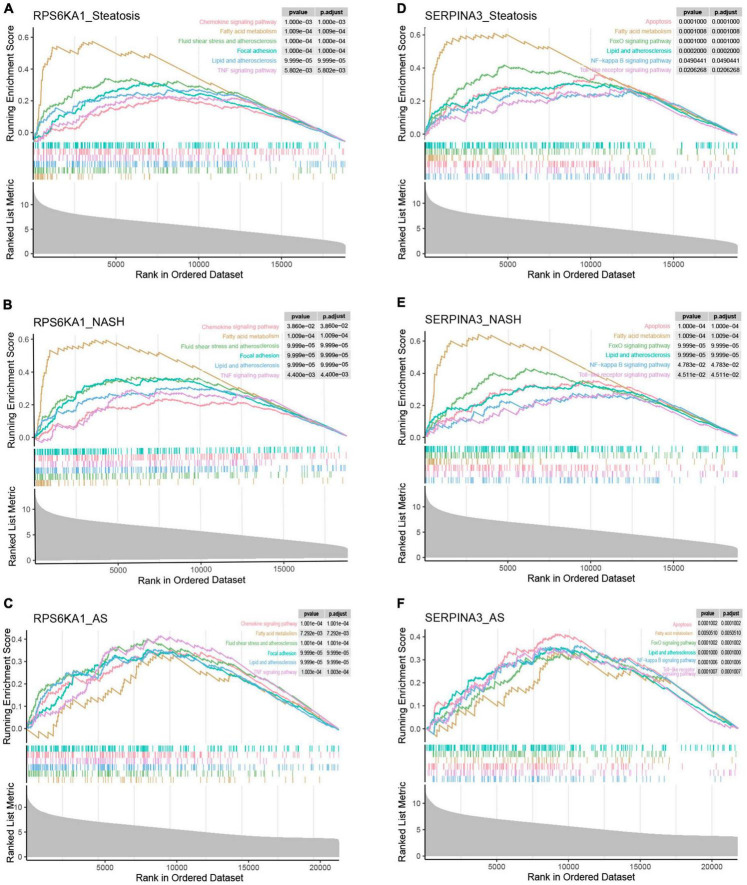
Single-gene GSEA of *RPS6KA1* and *SERPINA3*. Signaling pathways of *RPS6KA1* determined from single-gene GSEA in **(A)** steatosis, **(B)** NASH and **(C)** AS. Signaling pathways of *SERPINA3* determined from single-gene GSEA of in **(D)** steatosis, **(E)** NASH, and **(F)** AS.

To further discuss the potential role of the core genes *RPS6KA1* and *SERPINA3* in steatosis, NASH and AS, we analyzed pathways associated with a single gene using GSEA-KEGG. In the group with high expression, *RPS6KA1* might activate fatty acid metabolism, lipid and atherosclerosis, and TNF signaling, chemokine signaling, and focal adhesion pathways (Figures 8A-F), whereas *SERPINA3* might activate NF-κB signaling, Toll-like receptor signaling, and FoxO signaling pathways, apoptosis, fatty acid metabolism, lipid and atherosclerosis.

### Analysis of immune cell infiltration

**FIGURE 9:**
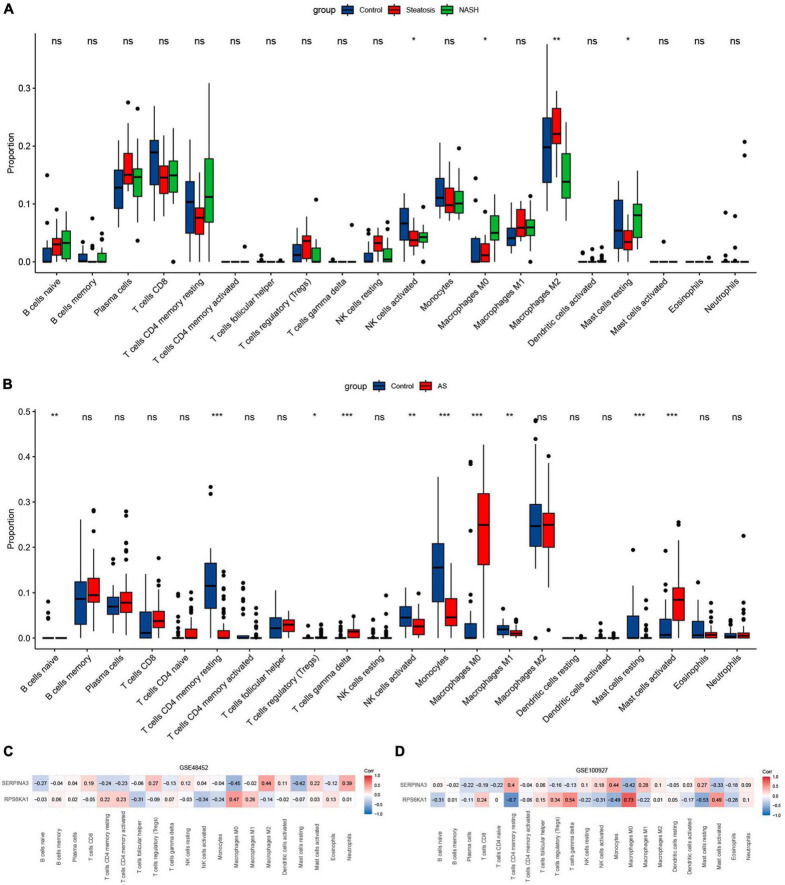
Immune infiltration assessed using CIBERSORT. Boxplots show comparisons of 22 immunocyte subtypes in **(A)** GSE48452, and **(B)** GSE100927. Heatmaps show correlation coefficients between hub genes and immunocyte subtypes in **(C)** GSE48452 and **(D)** GSE100927. *P < 0.05, **P < 0.01, and ***P < 0.001. ns indicates not significant (*p* > 0.05).

The results of immune cell infiltration in patients with using CIBERSORT revealed differences in the expression of activated NK cells, M0 macrophages, and resting mast cells among the steatosis, NASH, NAFLD, and AS groups compared with controls (Figures 9A, B). This trend was consistent, with significantly increased M0 macrophages and significantly decreased NK cell activation and resting mast cells. The results of correlation analyses between diagnostic *RPS6KA1* and *SERPINA3* gene expression and immune cells showed that *RPS6KA1* correlated positively with macrophages and *SERPINA3* correlated negatively with macrophages and mast cells in patients with NAFLD (Figure 9C), whereas *RPS6KA1* and *SERPINA3* respectively correlated positively and negatively with macrophages and mast cells in patients with AS (Figure 9D).

## Discussion

The principal purpose of this study was to determine crosstalk genes between NAFLD and AS using bioinformatics. We identified *RPS6KA1* and *SERPINA3* as the core crosstalk genes in NAFLD and AS, with far higher AUC values than numerous genes. Inflammatory activation participated in crosstalk between NAFLD and AS due to the co-expression of macrophages and mast cells, and that hub genes correlated to a greater degree with different immune cells.

The important identified gene, *RPS6KA1*, also called *P90RSK*, belongs to the RSK family of serine/threonine kinases (26). Upon activation induced by H_2_O_2_, *P90RSK* inhibits transcriptional activation of ERK5 and adjusts the subsequent expression of KLF2-eNOS and VCAM-1, thus inhibiting endothelial cells (ECs) inflammation and modulating the function of the ECs, which is responsible for atherosclerosis. However, this inhibitory effect was reversed by the *P90RSK* inhibitor (27). Blood flow disorder can easily progress to endothelial cell dysfunction, stimulate *P90RS0K* phosphorylation, induce SENP2 nuclear export, diminish SENP2 activity, enhance the sumoylation of ERK5 and tumor protein P53, and increase the expression of inflammatory and pro-apoptotic factors in ECs, which in turn decrease apoptosis and inflammatory responses that lead to their dysfunction; this ultimately results in the formation of atherosclerotic plaques (28–30). However, *P90RSK1* and *P90RSK2* gene knockout completely suppressed the appearance of such plaques. The mouse strain WT*P90RSK* -MTG that overexpresses *P90RSK* in bone marrow cells has far more atherosclerotic lesions and necrotic cores than non-transgenic littermate controls mice. In contrast, DN*P90RSK* -MTG mice expressed dominant-negative *P90RSK* in myeloid cells and have fewer obvious plaques and less developed necrotic cores. These results indicated that the activation of *RPS6KA1* (*P90RSK*) accelerates the formation of atherosclerotic plaques by influencing ECs function (31).

Direct investigations of *RPS6KA1* and NAFLD are limited. Currently, *RPS6KA1* plays an indeterminate role in chronic liver disease and might be involved in lipid metabolism and NAFLD. Inhibiting *RPS6KA1* and *STAT3* genes can impact the selective autophagic degradation of KLF3 mediated by CEBPB hat weakens adipogenesis (32).

Serine proteinase inhibitor A3 (*SERPINA3*) is also a key gene that belongs to the serpin superfamily (33). Most studies of *SERPINA3* have focused on tumors, and few have investigated NALFD and AS, whereas the mouse homologue *SERPINA3C* is related to two diseases in more studies. Plasma levels of the acute-phase protein *SERPINA3* increase substantially during inflammation (34). However, we found obviously diminished *SERPINA3* expression in patients compared with controls in the NAFLD and AS datasets, suggesting that *SERPINA3* has an anti-NAFLD (35) and anti-AS (36) effects. The anti-inflammatory role of *SERPINA3* is consistent with the results of its mouse homologue *SERPINA3C* (human homologue is *SERPINA3*) in NAFLD and AS.

Double knockout of *AP*O and *SERPINA3C* substantially increased lipogenic *SREBP1* and *SCD1* gene expression in the livers of mice fed with a high-fat diet. These mice developed lipid accumulation, and steatosis compared with only *APO* knockout. Serum AST and ALT levels are significantly increased in double-knockout mice, and macrophage infiltration and ECM content expression are also increased in their liver tissues (37). The increased expression of JNK and P65 proteins in the liver activates the JNK/NF-κB signaling pathway, which leads to enhanced expression of adhesion molecules and proinflammatory factor. All factors cause further liver inflammation and fibrosis, and significantly aggravate liver injury. Additionally, *SERPINA3C* can also resist hepatocyte necrotic apoptosis by inhibiting the β-catenin/Foxo1/TLR4 signaling pathway (37). A deficiency of *SERPINA3C* strengthens liver sensitivity to lipid toxicity and promotes necrotic apoptosis, which, in turn, promotes inflammation and fibrosis in NASH (38). In summary, *SERPINA3C* can inhibit the expression of genes associated with lipogenesis, curb lipogenesis and hepatocyte necrotic apoptosis through the JNK/NF-κB and Wnt/β-catenin signaling pathways, diminish inflammation and fibrosis in the liver, and inhibit the development of more severe NASH. This is consistent with our findings of significantly reduced *SERPINA3* expression in NASH in GSE89632, GSE48452, GSE58979 NAFLD datasets but did not significantly change in steatosis. Although we did not see similar results in the GSE130970 and GSE135251 validation sets, considering that there are more control samples in the GSE48452 and other datasets, and only 4 control samples in GSE130970, we also consider it as a core gene. Thus, *SERPINA3* might be a promising marker for a diagnosis of NASH syndrome and AS.

Otherwise, *SERPINA3C* has also been implicated in the development of AS. Thrombin activity is inhibited by *SERPINA3C*, which in turn weakens the inhibition of intracellular ERK1/2 and JNK phosphorylation, thereby declining the abnormal proliferation and migration of VSMCs. Excessive VSMC proliferation generates new intima and plaques, and facilitates the progression of atherosclerosis (39). These indicate that *SERPINA3C* is a novel thrombin inhibitor that protects against the development of atherosclerosis.

Atherosclerosis and NAFLD are the results of chronic inflammatory reactions, and pathological development mainly involves macrophages and mast cells (40). Immune infiltration analysis revealed an increased abundance of macrophages and mast cells in patients with AS and those with NAFLD. Accumulated mast cells in plaques promotes the progression of atherosclerosis by secreting pro-inflammatory factors and chemokines (41). Macrophages absorb low-density lipoprotein particles that have been modified by lipases and proteases and are trapped in the intima of blood vessels to form foam cells, which comprise the hallmark of early plaque formation (42). The continual accumulation of foam cells in the vascular intima aggravates local inflammatory responses by constantly secreting inflammatory factors and chemokines. Activation of *P90RSK* in macrophages guides the expression of pro-inflammatory genes like *TNF-α*, which in turn facilitates atherosclerosis. Moreover, *P90RSK* overexpression in macrophages is involved in atherosclerotic and necrotic core formation.

Our results indicated that lipid metabolism, endothelial dysfunction, and inflammatory responses play vital roles in NAFLD and AS pathogenesis. Endothelial cell dysfunction is an initial stage of atherosclerosis (43, 44). The abundance of ECs expressing cell adhesion molecules such as E-selectin, increases upon contact with a multitude of hazardous factors for atherosclerosis. These adhesion molecules then release chemokines that induce the recruitment and infiltration of monocytes and lymphocytes. The adhesion of monocytes and lymphocytes to ECs constitutes a proportion of atherosclerotic lesions, augments the recruitment of white blood cells and activates proinflammatory factors in ECs. This leads to inflammation that ultimately exacerbates atherosclerosis.

During the progression from simple steatosis to more severe NASH, Endothelial dysfunction and inflammation are important role (10, 45). Hepatic sinusoidal endothelial cells activate NF-κB signaling through TLR9 to reinforce secretion of CCR2 and CXCR4, recruit macrophages to inflammatory sites, and release inflammatory factors during this period. These inflammatory factors might cause vascular endothelial cell dysfunction, which in turn results in atherosclerosis (46, 47).

As both NAFLD and AS are manifestations of end-organ damage in metabolic syndrome, they have several similarities. However, the common mechanisms of NAFLD with different stages and AS has not been explored until now. Based on the WGCNA and MLAs, we identified characteristic genes that will help to further elucidate the mechanisms of action that are common to NAFLD with different stages and AS of progression. Our study was retrospective, and further experimental and clinical data are required to confirm our results. We will collect cases of NAFLD combined with AS in clinical practice, which will be our future research. The expression and diagnostic models based on two signature genes functioned well in the external validation sets for NAFLD with different stages and AS of progression, which increased confidence in our results. We revealed new biomarkers of an association between NAFLD with different stages and AS of progression.

## Data availability statement

The original contributions presented in the study are included in the article/Supplementary material, further inquiries can be directed to the corresponding author.

## Author contributions

LW: Writing−original draft, Data curation, Formal analysis, Funding acquisition, Methodology, Software, Visualization. WH: Writing−original draft, Formal analysis, Methodology. XW: Formal analysis, Writing−original draft. JW: Writing−review and editing. XW: Writing−review and editing. DW: Writing−review and editing. YW: Writing−review and editing.

## Footnotes

1
https://www.ncbi.nlm.nih.gov/geo/
