# Supplementary figures and images for: Potential diagnostic markers shared between non-alcoholic fatty liver disease and atherosclerosis determined by machine learning and bioinformatic analysis
Source: Front Med (Lausanne). 2024 Mar 28;11:1322102. doi: 10.3389/fmed.2024.1322102 (PMC11007109; doi:10.3389/fmed.2024.1322102)

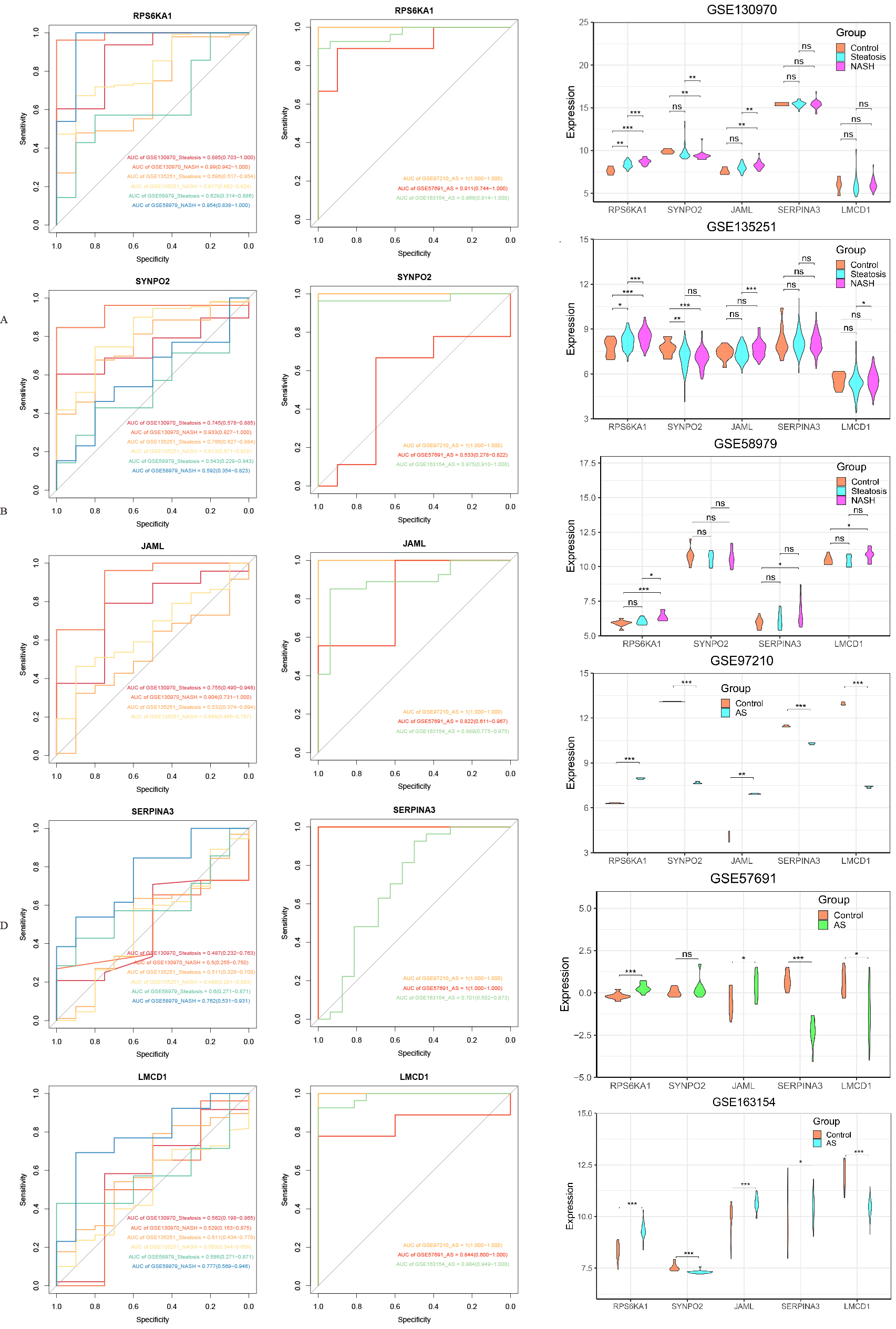

Supplement: Supplementary file 1 [file Data_Sheet_1.ZIP › Supplementary Material/Supplementary Figure4.jpg]
